# The Use of Exercise Echocardiography in the Evaluation of Mitral Regurgitation

**DOI:** 10.2174/157340309789317841

**Published:** 2009-11

**Authors:** Kibar Yared, Kaitlyn My-Tu Lam, Judy Hung

**Affiliations:** Department of Medicine, Division of Cardiology, Massachusetts General Hospital, Harvard Medical School, Boston, Massachusetts, USA

**Keywords:** Stress echocardiography, mitral valve, mitral regurgitation, cardiac resynchronization therapy.

## Abstract

Mitral regurgitation (MR) is the second most common valvular disease in western countries after aortic stenosis. Optimal management of patients with MR depends on the etiology of the regurgitation and is based predominantly on left ventricular function and functional status. Recent outcome studies report high risk subsets of asymptomatic patients with MR, and practice guidelines underscore the importance of a well-established estimation of exercise tolerance and recommend exercise testing to objectively assess functional status and hemodynamic factors.

## INTRODUCTION

Mitral regurgitation (MR) is the second most common valve disease in western countries, after aortic stenosis, representing 32% of single native left-sided valve disease in a recent European survey [1]. MR results from several heterogeneous conditions and its many causes include disorders of the valve leaflets, mitral annulus, chordae tendinae, papillary muscles and the left ventricle. Primary MR is defined as MR resulting from organic valvular pathology such as prolapse, rheumatic change or that due to endocarditis. Secondary MR results from ischemic or myopathic changes to the left ventricle leading to incomplete closure of the mitral leaflets. In the latter conditions, leaflet morphology is relatively normal.

Exercise echocardiography plays an important role in the evaluation and management of chronic mitral regurgitation. It can assist in the evaluation of symptoms, more fully assess the mechanism and severity of regurgitation, determine functional capacity, and assess contractile reserve to help optimize the timing of surgical intervention. In addition, exercise echocardiography has been beneficial in assessing the response of MR to cardiac resynchronization therapy.

This review will summarize the clinical utility of exercise echocardiography in chronic MR, examining both primary and secondary MR.

## PATHOPHYSIOLOGY

The regurgitant orifice in MR serves as an “escape valve”, decreasing the impedance to and enhancing left ventricular emptying. The volume of MR flow depends on both the size of the regurgitant orifice and the (reverse) pressure gradient between the left ventricle (LV) and the left atrium (LA) [2]. Both the orifice size and the pressure gradient  are  dynamic [3-5]. Increases  in  both  preload  and afterload and depression of contractility increase left ventricular size, enlarge the mitral annulus and displace the papillary muscles apically thereby increasing the regurgitant orifice. The pressure gradient between the LV and LA can increase with increases in LV afterload, preload as well as contractility.

In chronic MR, volume overload of the left ventricle results in an increase in LV end-diastolic volume (LVEDV). The increase in LVEDV allows augmentation of total stroke volume, while maintaining forward stroke volume near normal. Ejection indices such as ejection fraction and fractional shortening are initially supra-normal due to the low impedance of the outflow circuit; these indices can appear “normal” even after contractile function has been impaired [6].

Although the compensatory mechanisms are effective, the prolonged volume overload ultimately leads to myocardial dysfunction. Consequently, end-systolic volume rises, and ejection fraction and stroke volume decline. Therefore, surgical intervention is usually recommended prior to the onset of LV dysfunction and is important in preventing further deterioration of systolic function and improve survival [7].

The hemodynamic response to exercise in MR depends on the change in the severity of the regurgitant lesion and the ability of the left ventricle to meet increases in demands on workload. These two factors interact in complex ways. The effective regurgitant orifice (ERO) can be relatively fixed throughout systole such as in rheumatic mitral valve disease. On the other hand, in ischemic MR, and occasionally in cases with flail mitral leaflets [4], the ERO can be dynamic and may vary with the response of the ventricle to exercise. The response of the ventricle to exercise, another determinant of the response of MR to exercise, depends on its contractile state at rest, as well as its contractile response to exercise.

Risk stratification using exercise echocardiography, especially in asymptomatic patients, becomes important not only in accurate quantification and characterization of the etiology of MR, but also in guiding therapy.

## PRIMARY MITRAL REGURGITATION

### Symptomatic Patients

**Fig. (1):**
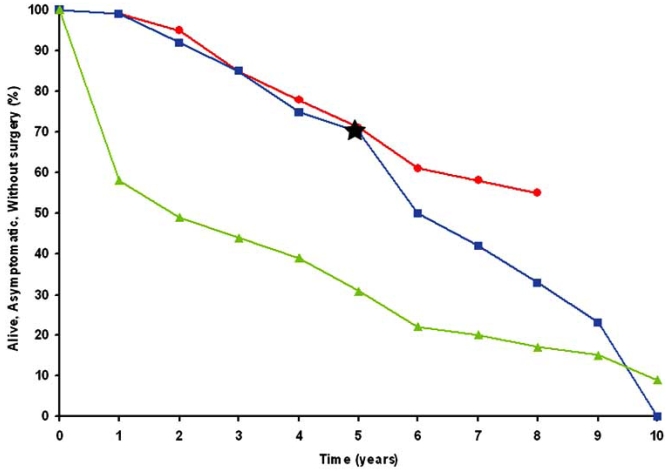
Four series examining the natural history of patients with severe MR including a series of patients with flail mitral leaflets reported by Ling and associates (green triangles), many of whom were symptomatic, had atrial fibrillation, or had evidence of left ventricular (LV) dysfunction, and three series reported by Rosen *et al*. (blue squares), Sarano *et al*. (black star) and Rosenhek *et al*. (red circles) in patients who initially were asymptomatic with normal LV function. Although the patients with flail leaflets had a steeper initial attrition rate, all series demonstrated that patients with severe MR have a high likelihood of developing symptoms or other indications for surgery over the course of 6 to 10 years. Modified from Otto CM, Bonow RO. Valvular Heart Disease. In: Libby P, Bonow RO, Mann DL, *et al*., Eds. Braunwald's Heart Disease. A Textbook of Cardiovascular Medicine. 8^th^ ed. Philadelphia: Saunders Elsevier 2007: 1664.

The most recent ACC/AHA guidelines for the management of valvular disease [8] include, as a class I indication, surgical intervention once patients develop symptoms associated with severe MR in the setting of normal LV function. Among symptomatic patients with flail leaflets, Ling and Enriquez-Sarano [9] reported an annual mortality rate of 6.3%; at 10 years 90% had died or had undergone surgical correction (Fig. **1**). This latter series included many patients who had left ventricular dysfunction or atrial fibrillation and thus might have been considered to be at a higher risk.

With the current guidelines, reasonable surgical candidates who develop symptoms related to MR should undergo surgical correction. However, symptom status can be subjective and variable and it is sometimes difficult to relate those symptoms to severe mitral regurgitation. Patients also often have other co-morbidities that may be contributing to their symptoms. Due to the morbidity and mortality associated with severe MR and the potential benefit of surgical intervention, it is important to differentiate symptoms caused by mitral regurgitation from those of other causes. Differentiating the symptoms of chronic obstructive pulmonary disease (COPD), for example, from those of MR can be extremely challenging. Approaches involving combined cardio-pulmonary function testing during exercise may help distinguish between pulmonary and cardiovascular etiologies of dyspnea [10]. Exercise echocardiography may also unmask and clarify subclinical symptoms. The observation of limiting symptoms with worsening MR during exercise echocardiography suggests that the patient’s symptoms are to due to exercise-induced MR.

In symptomatic patients with a clinical picture suspicious for severe MR, but not evident on the resting echocardiogram, exercise echocardiography demonstrating worsening MR helps correlate the pathology with the patient’s symptoms [11]. Similarly, in a study of patients with poor exercise tolerance and rheumatic mitral valve disease with only a mild degree of MR, exercise echocardiography has been helpful in elucidating the origin of the symptoms by provoking severe MR in some patients and significant mitral stenosis in others, with associated pulmonary hypertension [12]. These findings have been applied to patients without rheumatic heart disease.

### Asymptomatic Patients

**Fig. (2):**
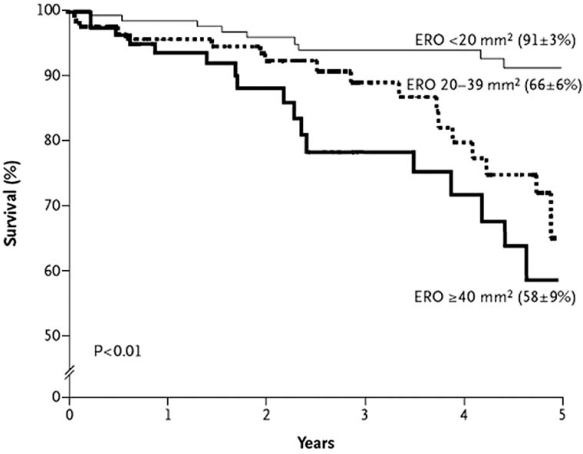
Kaplan–Meier estimates of the mean (± standard error) rates of overall survival among patients with asymptomatic mitral regurgitation undergoing medical management, according to the effective regurgitant orifice (ERO). Values in parentheses are survival rates at five years. From Enriquez-Sarano M, Avierinos J-F, Messika-Zeitoun D, *et al*. Quantitative Determinants of the Outcome of Asymptomatic Mitral Regurgitation. N Engl J Med 2005; 352(9): 875-883.

Among asymptomatic patients with initially normal left and right ventricular ejection fractions, severe MR is associated with a high rate of symptoms or left ventricular dysfunction requiring surgery. Mitral valve surgery (repair or replacement) during a 5-year follow-up was independently associated with a reduced risk of death [13]. In 102 asymptomatic patients with normal LV function, the 5-year combined incidence of atrial fibrillation, heart failure, or cardiovascular death was 42 ± 8%. In these patients, surgery also reduced rates of cardiovascular death and heart failure together [13]. In fact, mitral valve surgery was nearly unavoidable over the course of 10 years [14]. Recent studies have also shown that the clinical course of initially asymptomatic primary MR is not necessarily benign [15, 16]. These studies have shown that patients with medically managed asymptomatic MR and an effective regurgitant orifice of > 40 mm^2^ had an excess risk of death and cardiac events (Fig. **2**). In addition, cardiac surgery markedly reduced the risk of heart failure and death. Conversely, watchful waiting of asymptomatic patients with severe MR until development of symptoms, LV dysfunction or dilatation, or pulmonary hypertension has recently been advocated [17]. 132 consecutive patients with severe degenerative MR, due to flail leaflet or prolapse, were followed up for 62 ± 26 months. During the follow-up period, 38 patients developed an indication for surgery based on ACC/AHA guidelines. Survival free of any indication for surgery was 92% at 2 years, 78% at 4 years, 65% at 6 years, and 55% at 8 years. Patients with a flail leaflet tended to develop criteria for surgery slightly but, not significantly, earlier than those with mitral valve prolapse. Overall survival was not statistically different from expected survival either in the total group or in the subgroup of patients with flail leaflet.

With exercise, mitral regurgitation that is non-holosystolic can appear *de novo* or even increase in duration and severity. Stoddard *et al.* [18] studied 94 patients with mitral valve prolapse and no MR. After symptom-limited exercise using supine bicycle ergometry, 30 patients (32%) developed transient MR. At peak stress, there was no significant difference in ejection fraction between those who developed MR and those who did not. The only hemodynamic variable that differed between the two groups was peak systolic blood pressure, with a greater increase in those who developed MR. During the follow-up period of 38 months, the patients with exercise-induced MR had a six-fold greater incidence of cardiovascular morbid events, particularly syncope and congestive heart failure. Exercise-induced MR and a prior history of syncope were the only independent predictors of a subsequent cardiovascular event or syncope and exercise-induced MR was the only independent predictor of congestive heart failure. Patients with mitral valve prolapse without MR at rest and no leaflet thickening are usually considered at low risk for morbid events. The study by Stoddard *et al.* showed that more than 30% of these “low risk” patients have exercise-induced MR and are at a higher risk for morbid events than usually expected [18]. Exercise echocardiography therefore can have important prognostic value in patients with mitral valve prolapse.

Stress echocardiography has been evaluated in the setting of mitral regurgitation secondary to rheumatic heart disease. Symptoms can often appear to be out of proportion to the degree of valvular pathology. Tischler *et al.* [12] performed echocardiography prior to and after exercise in 14 patients with symptomatic, mild, mixed rheumatic mitral valve disease to ascertain the actual cause of symptoms. These patients had a normal ejection fraction and no more than mild mitral regurgitation or mild mitral stenosis. Diastolic mitral gradients increased with exercise, but an unexpected finding was the development of severe mitral regurgitation in five patients (36%). Patients who developed mitral regurgitation trended toward a diminished exercise capacity. Although decreased exercise capacity was not clearly correlated with the patients’ symptoms, it was felt likely that the acute onset of severe mitral regurgitation was a factor in the limitation of exercise.

Left ventricular contractile function can be impaired even in the presence of a normal ejection fraction in patients with chronic MR because of altered loading conditions [6, 19,  20]. Exercise echocardiography can unmask latent or subclinical LV dysfunction in patients in whom the LV is compensated at rest. An inability to increase the ejection fraction or reduce the end-systolic volume with stress reflects the presence of an impaired contractile reserve, and have both been shown to be reliable early markers for progressive deterioration in myocardial contractility [21].

Lee *et al.* evaluated functional and prognostic implications of LV contractile reserve in patients with isolated severe MR pre- and post-mitral valve replacement as well as in those being treated medically [22]. Contractile reserve was defined as the difference between the resting and post-exercise EF. An impaired contractile reserve (EF increment post-exercise ≤ 4%) predicted not only the development of late post-operative LV dysfunction (EF < 50%) and morbidity in surgically treated patients but also the occurrence of cardiac events (congestive heart failure and new onset atrial fibrillation) and progressive deterioration of LV function in the medically treated patients. Conversely, an intact contractile reserve (EF increment post-exercise > 4%) predicts preservation of LV function and a favorable clinical outcome irrespective of whether patients were treated medically or surgically. Additionally, evaluation of contractile reserve had an incremental value over rest LV end-systolic volumes in predicting late post-operative LV dysfunction. An important limiting factor in this study is the inevitable variability encountered when measuring post-exercise end-systolic and end-diastolic volumes – the manner in which EF was derived. Accurate tracing of endocardial borders is very challenging in patients with poor acoustic windows and technically difficult studies, and introduces important inter-observer variability that limits the general applicability of such a technique.

A similar study used exercise echocardiography prior to mitral valve surgery to identify those patients with asymptomatic, chronic severe MR who were at risk of developing postoperative LV dysfunction [23]. A postoperative ejection fraction of < 50% was best predicted by a pre-operative exercise end-systolic volume index > 25 ml/m^2^, an exercise ejection fraction < 68% and an increase in ejection fraction with exercise of < 4% (Table **1**) [23].

Evaluation of right ventricular function and pulmonary artery pressure, especially with exercise, can aid in deciding to intervene on patients with severe MR (ACC/AHA Class IIa indication). In patients with mitral valve prolapse, the change in right ventricular ejection fraction with exercise was the only predictor of the need for surgical intervention over 4.7 years of follow-up [9]. Hochreiter *et al.* studied asymptomatic and symptomatic patients with significant mitral regurgitation due to diverse etiologies (most with LV ejection fraction < 50%). Right ventricular ejection fraction both at rest and during exercise was slightly more predictive of long term mortality than were left ventricular ejection fraction during rest and exercise, symptoms and treadmill exercise time during follow-up [24]. Although assessment of left ventricular performance at rest and post-exercise provides a certain amount of prognostic information, the assessment of right ventricular function post-exercise has shown utility in prognostication in patients with chronic MR, and suggests a potential role for such an assessment in the selection of patients for operation, irrespective of symptom status. This type of recommendation, however, will require further testing.

The onset of resting pulmonary hypertension (> 50mm Hg) secondary to severe mitral regurgitation is considered an indication for surgery, for, if left untreated, it is associated with a greater severity of mitral regurgitation and higher operative morbidity and mortality [25]. Furthermore, pulmonary hypertension increases the likelihood of significant tricuspid regurgitation and right ventricular dysfunction. Increased systolic pulmonary artery pressure (estimated from the tricuspid regurgitant jet velocity) during exercise has been used in an uncontrolled setting to identify occult left ventricular dysfunction and need for mitral surgery [26]. The degree of mitral regurgitation is one the major factors contributing to pulmonary hypertension, and is associated with higher morbidity and mortality in patients with LV dysfunction [27, 28]. However, even in the presence of normal resting pulmonary artery pressures, measured systolic pulmonary artery pressures of > 60 mmHg post-exercise is an indication for mitral valve intervention [8]. Exercise echocardiography, therefore, can play an important role even in the presence of normal resting pulmonary pressures.

Although exercise capacity is an important parameter of quality of life and provides prognostic information, cardiac determinants affecting it have not been clearly defined in patients with chronic MR. It is well known that LV diastolic function is an important determinant of exercise capacity in normal individuals and in patients with various cardiac disease [29, 30]. To address the contribution of LV diastolic function to exercise capacity, the group of Kim *et al.* studied 32 patients with severe MR and normal LV ejection fraction. They observed that the early diastolic mitral annulus velocity (E’) and pulmonary vein A-wave peak velocity (PVa), both accepted surrogate estimates of LV diastolic function, showed a significant correlation with maximal exercise time [31]. This study also corroborated earlier findings that LV ejection fraction and fractional shortening were poor predictors of exercise capacity [32, 33]. From these results, the authors concluded that LV systolic parameters were not significant determinants of exercise capacity in patients with chronic MR and a preserved EF. Rather, the measurement of E’ and PVa was more useful in identifying patients with chronic MR and poor exercise capacity.

In chronic, severe mitral regurgitation, once patients develop symptoms or LV dysfunction, the decision to intervene becomes straightforward. However, decision-making is more challenging in patients with vague, atypical symptoms. It is important to accurately, and in a timely fashion, identify symptoms related to chronic MR as well as occult LV dysfunction. The combination of stress testing with echocardiography can detect and quantify occult symptoms as well as worsening mitral regurgitation. Stress echocardiography, therefore, provides a comprehensive and cost-effective evaluation of patients with primary mitral regurgitation that combines functional, diagnostic, and prognostic information.

## ISCHEMIC MITRAL REGURGITATION

**Fig. (3):**
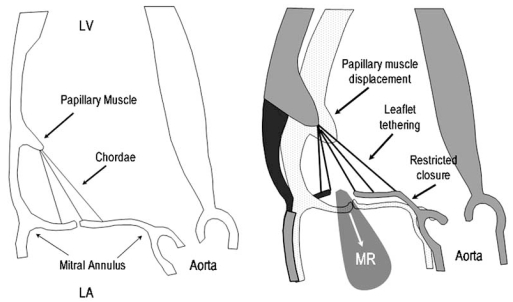
Left panel: Schematic depicting the balance of forces applied to the mitral valve. Right panel: Demonstration of the potential effect of a posterior and lateral shift of the posterolateral papillary muscle, combined with annular dilatation, restraining the mitral leaflets from meeting each other and causing MR. Adapted from Otsuji Y, Handschumacher MD, Schwammenthal E, *et al*. Insights from three-dimensional echocardiography into the mechanism of functional mitral regurgitation: direct in vivo demonstration of altered leaflet tethering geometry. Circulation. 1997; 96(6): 1999-2008.

Ischemic mitral regurgitation (IMR) results from restricted leaflet closure secondary to tethering of the mitral leaflets as a consequence of an infarcted, thinned ventricle. IMR is characteristically dynamic and sensitive to changes in ventricular size, shape, and loading that restrict closure of the mitral leaflet [34, 35]. In ischemic left ventricular dysfunction, the mitral annulus may dilate and the inferoposterior wall bulges outward, displacing the attached papillary muscles apically and outward [36]. The leaflets, tethered at both ends, cannot close effectively and are restrained within the left ventricle with resultant regurgitation (Fig. **3**). Further studies have corroborated that the degree of ischemic MR at rest in patients with systolic dysfunction is determined by local (apical and posterior displacement of papillary muscles) and not global LV remodeling [37, 38]. 

IMR is a dynamic phenomenon, its severity varying depending on loading conditions [36, 39]. Often patients might complain of dyspnea on exertion, out of proportion to the amount LV dysfunction or MR at rest. Patients with exercise-induced increases in IMR severity incur limited stroke volume adaptation during exercise and this contributes, in part, to the limitation in exercise capacity [40]. Exercise-induced changes in regurgitant volume and in systolic pulmonary pressure are larger in patients who stop their exercise because of dyspnea as compared with those who stop for fatigue [41]. Exercise echocardiography can be extremely useful in qualifying and quantifying a change in the amount of mitral regurgitation seen during exercise; information that cannot be obtained by resting echocardiography. 

Pierard and Lancellotti showed that patients with known ischemic LV dysfunction who present with acute pulmonary edema not due to myocardial ischemia demonstrate large exercise-induced increases in mitral regurgitation and related pulmonary pressures [42]. Exercise testing coupled with quantitative Doppler echocardiography could be useful for patients with left ventricular systolic dysfunction in whom acute pulmonary edema develops without an obvious cause. A mild degree of mitral regurgitation at baseline can be associated with large dynamic changes, explaining the clinical spectrum from exertional dyspnea to the occurrence of acute pulmonary edema.

IMR has also been shown to worsen with worsening LV function during exercise [43]. Annular enlargement [44], persistent leaflet tethering [38], LV dilation, sphericalization [45], and dysfunction have all been proposed as determinants of ischemic MR. However, in a study of 70 patients in the chronic post-MI phase, with LV ejection fraction < 45% and at least mild MR undergoing semi-supine exercise Doppler echocardiography, ERO at rest did not predict changes in the ERO during exercise [46]. As well, the degree of exercise-induced increase or decrease in MR appeared to relate to local LV remodeling and mitral valvular deformation but not to changes in global LV function, as mentioned previously. Similarly, in a more recent study, changes in ERO were unrelated to the severity of ischemic MR at rest, as well as to the severity of LV dysfunction [47]. Exercise-induced changes in MR were related to changes in wall motion score index, as well as to changes in end-systolic sphericity index. The strongest predictors of exercise-induced changes in ischemic MR in patients with LV dysfunction due to prior MI were exercise-induced changes in mitral deformation (Table **2**). 

During exercise, the magnitude of changes in IMR is widely different from one patient to another and does not correlate with the degree of MR at rest. Most patients exhibit small increases in the amount of IMR, whereas others have either a large rise or a significant decrease in ERO [46]. The demonstration of an exercise-induced reduction in IMR as a result of recruitable function in the basal part of the inferior wall can be used to predict the reduction in IMR post-revascularization of the coronary artery serving that specific territory [47]. Otherwise, in the case of increased IMR with exercise, perhaps a combination of revascularization and mitral valvular surgery may be necessary.

Ischemic mitral regurgitation may exhibit a broad range of severity and conveys a dismal prognosis [48-50]. The increased mortality risk relates not only to the presence, but also more importantly to the quantified degree of IMR [41, 51]. In patients with ischemic MR and left ventricular dysfunction, quantitative assessment of exercise-induced changes in the degree of MR provides independent prognostic information. Significant exercise-induced increases in MR unmask patients at high risk for morbidity and mortality [51-53]. Predictors of cardiac death included an increase in ERO by ≥ 13 mm^2^ during exercise, an ERO > 20mm^2^ at rest [51], greater LV volumes at rest and lack of contractile reserve during exercise [52].

The use of exercise echocardiography is becoming a more valuable tool in the evaluation of ischemic MR. It can be used to quantify, and determine the reason for limitations in, exercise capacity. As well, it’s utility in testing the dynamic component of IMR in patients with chronic ischemic LV dysfunction presenting with unexplained dyspnea or acute pulmonary edema cannot be ignored. Exercise echocardiography has been shown to be useful in the evaluation and risk stratification of patients with IMR.

## MITRAL REGURGITATION AND CARDIAC DYSSYNCHRONY

Mitral regurgitation is seen in over half of all patients with symptomatic heart failure and is an independent predictor of mortality [54]. Non-ischemic functional mitral regurgitation (FMR) in dilated cardiomyopathy occurs in the setting of incomplete closure of a structurally normal mitral valve and progressive ventricular remodeling associated with left ventricular dysfunction and dilation. Many patients with chronic LV dysfunction develop limiting symptoms only with activity. However, unfortunately many of our objective assessments of their pathology occur at rest. Exercise capacity in heart failure patients depends on the efficiency of delivery of oxygen to the exercising muscles. Thus in addition to cardiac output, peripheral factors such as skeletal muscle function, endothelial cell function and autonomic nervous system function play an essential role in exercise tolerance. Multiple studies have demonstrated an increase in functional MR with exercise in patients throughout the range of heart failure severity associated with a decrease in stroke volume [40, 55]. However, the worsening of FMR did not induce a deterioration of the anaerobic thresholds which is determined by peripheral factors [55]. 

Cardiac resynchronization therapy (CRT) improves exercise tolerance in addition to providing symptomatic and mortality benefits in patients with severe heart failure refractory to  medical  therapy [56-58]. The  potential  mechanisms for CRT success are related mainly to optimized LV filling [59], synchronized LV electromechanical coupling resulting in an improvement in LV myocardial performance [59], and a reduction of mitral regurgitation [60, 61] (Fig. **4**). The cardiac structural changes associated with CRT include not only volumetric improvement but also true reverse LV structural remodeling, evidenced by progressive reduction in LV mass and restoration of regional wall symmetry [62], improved left ventricular function [59] and improved cardiac synchrony. Multiple studies have shown CRT improves resting MR in the acute and chronic settings with a corresponding worsening in the degree of MR upon interruption of therapy [63-67]. 

**Fig. (5):**
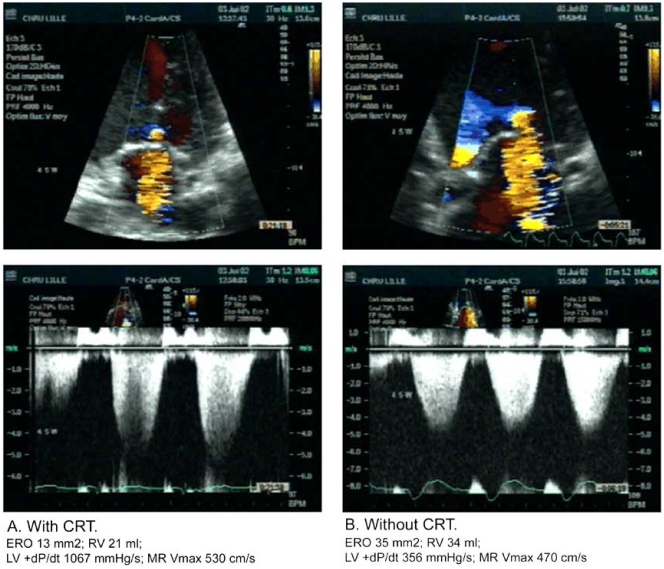
Colour flow (top) and continuous wave (bottom) Doppler echocardiographic recordings in a representative patient at maximal exercise with (Panel A) and without (Panel B) cardiac resynchronisation therapy (CRT). Left ventricular (LV) dP/dt and maximum mitral regurgitation velocity (MR Vmax) were reduced without CRT. ERO, effective regurgitant orifice area; RV, regurgitant flow volume. From Ennezat PV, Gal B, Kouakam C, *et al*. Cardiac resynchronisation therapy reduces functional mitral regurgitation during dynamic exercise in patients with chronic heart failure: an acute echocardiographic study. Heart (British Cardiac Society) 2006; 92(8): 1091-1095.

There are varying results describing the effect of CRT on exercise-induced MR prior to the occurrence of LV remodeling. Ennezat *et al.* showed that CRT attenuated the increase in mitral ERO and regurgitant volume during exercise among 21 patients undergoing symptom-limited exercise stress testing; a phenomenon attributed to an increase in LV contractility (Fig. **5**) [64]. Conversely, Madaric *et al.* did not show an improvement in exercise-induced MR within the 1^st^ week of implantation. However, the attenuation of exercise-induced MR did occur in parallel with LV remodeling at 3 months post-implantation [66]. The varying results may be explained by the response to resynchronization of the two left ventricular papillary muscles [67, 68]. 

**Fig. (6):**
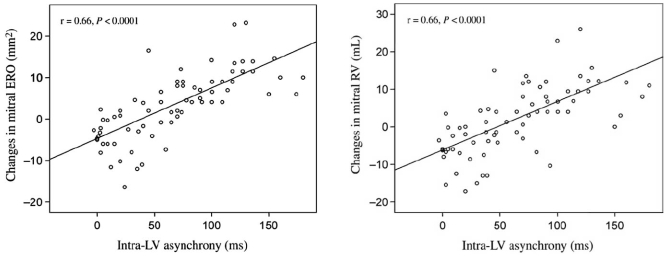
Relationships between intra-LV asynchrony and exercise-induced changes in mitral ERO (left panel) and regurgitant volume (RV) (right panel). From Ennezat PV, Marechaux S, Le Tourneau T, *et al*. Myocardial asynchronism is a determinant of changes in functional mitral regurgitation severity during dynamic exercise in patients with chronic heart failure due to severe left ventricular systolic dysfunction. Eur Heart J 2006; 27(6): 679-683.

LV asynchronism, as measured by Doppler tissue imaging at rest, substantially contributes to worsening of MR during dynamic exercise in patients with CHF due to LV systolic dysfunction (Fig. **6**) [69] Ypenburg *et al.* studied 25 patients who showed an immediate improvement in MR after CRT was accompanied by an improvement in mitral deformation indices (tenting area, coaptation height and annular contraction) and synchrony between the two LV papillary muscles. Acute loss of resynchronization after interruption of CRT lead to acute recurrence of MR and worsening in mitral deformation indices [67]. 

D’Andrea *et al.* described a series of 60 patients with idiopathic dilated cardiomyopathy and QRS interval < 120 ms who were submitted to supine bicycle exercise Doppler echocardiography and cardiopulmonary exercise testing [70]. Dynamic LV dyssynchrony unmasked by exercise was found in more than 50% of the patients. Increased LV dyssynchrony during exercise was independently associated with increased functional MR, reduced exercise capacity, and lower LV stroke volume at peak exercise. This study illustrates the concept of dynamic dyssynchrony, a notion that needs further investigation to clarify its mechanisms and clinical implications.

There is a close correlation between exercise-induced changes in LV dyssynchrony and functional MR. Acute changes in MR have been observed with the induction and interruption of CRT. Initiation of CRT acutely reduces MR severity at rest and its dynamic component during exercise well before the occurrence of significant LV inverse remodeling [65, 71]. In addition, the interruption of biventricular pacing is associated with an acute increase in MR [63]. Certain clinical implications of dynamic MR have already been reported [42]. The demonstration of worsening dyssynchrony, and consequent increased MR, with exercise deserves further study and may change the criteria for selecting patients for CRT [72]. 

## SUMMARY

In summary, exercise echocardiography provides useful data in patients with mitral regurgitation. Information that can be acquired with this modality include:

- An assessment of functional capacity;
- A quantification of change in MR severity during exercise;
- An assessment of contractile reserve of the left ventricle;
- An evaluation of the response of the right ventricle, especially with worsening MR during exercise;
- Obtaining prognostic information.

## Figures and Tables

**Fig. (4). An example of acute reduction in mitral regurgitation by color Doppler before (left) and immediately after (right) cardiac resynchronization therapy (CRT).**
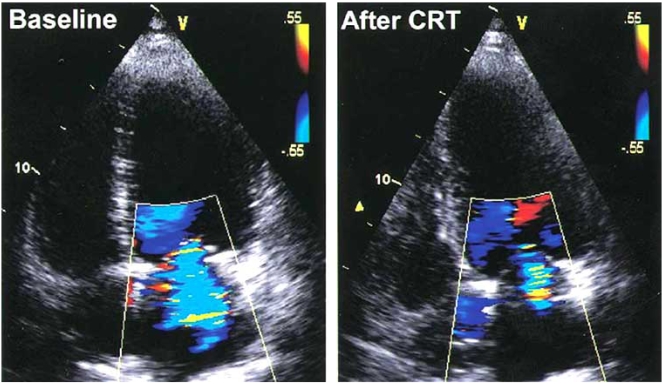
From Kanzaki H, Bazaz R, Schwartzman D, *et al*. A mechanism for immediate reduction in mitral regurgitation after cardiac resynchronization therapy: Insights from mechanical activation strain mapping. J Am Coll Cardiol 2004; 44(8): 1619-1625.

**Table 1:** Sensitivity and Specificity of Diagnostic Cutoff Values of Preoperative Rest and Exercise Indexes of Left Ventricular Function in Predicting Early Postoperative Left Ventricular Dysfunction

Variable	Optimal Diagnostic Cut-Off	Specificity (%)	Sensitivity (%)
ESVI_exercise_	25 cm^3^/m^2^	83	83
EF_exercise_	68%	80	81
ΔEF_exercise_	4%	75	79
LV dP/dt	1000 mmHg/s	73	65
ESWS_rest_	52.4 x 10^3^ dynes/cm^2^	65	64
ESVI_rest_	29 cm^3^/m^2^	63	66
EF_rest_	66%	51	67

ESVI_exercise_, end systolic volume index immediately after exercise; EF_exercise_, ejection fraction immediately after exercise; ΔEF_exercise_, change in ejection fraction with exercise; LV dP/dt, peak rate of change in left ventricular pressure; ESWS_rest_, end systolic wall stress at rest; ESVI_rest_, end systolic volume index at rest; EF_rest_, ejection fraction at rest. From Leung et al. [23].

**Table 2:** Predictors of Exercise-Induced Changes in Mitral Regurgitation

	Rest	Exercise	*p*-value	Δ Value	*p*-value
Mitral annular dimension (cm)	3.7 ± 0.2	3.8 ± 0.3	0.014	0.13 ± 0.32	0.0001
Coaptation distance (cm)	1.5 ± 0.1	1.9 ± 0.6	0.0001	0.41 ± 0.55	0.0001
Tenting area (cm^2^)	3.5 ± 0.3	4.4 ± 0.9	0.0001	0.87 ± 1.34	0.0001

Adapted from Giga *et al*. [47].

## References

[1] Iung B, Baron G, Butchart EG (2003). A prospective survey of patients with valvular heart disease in Europe: The Euro Heart Survey on Valvular Heart Disease. Eur Heart J.

[2] Carabello BA (2003). Progress in mitral and aortic regurgitation. Curr Probl Cardiol.

[3] Neilan TG, Ton-Nu T-T, Kawase Y (2008). The progressive nature of chronic mitral regurgitation and the role of tissue doppler derived indices. Am J Physiol Heart Circ Physiol.

[4] Yellin EL, Yoran C, Sonnenblick EH, Gabbay S, Frater RW (1979). Dynamic changes in the canine mitral regurgitant orifice area during ventricular ejection. Circ Res.

[5] Yoran C, Yellin EL, Becker RM (1979). Dynamic aspects of acute mitral regurgitation: effects of ventricular volume, pressure and contractility on the effective regurgitant orifice area. Circulation.

[6] Wisenbaugh T (1988). Does normal pump function belie muscle dysfunction in patients with chronic severe mitral regurgitation?. Circulation.

[7] Enriquez-Sarano M, Tajik AJ, Schaff HV (1994). Echocardiographic prediction of survival after surgical correction of organic mitral regurgitation. Circulation.

[8] Bonow RO, Carabello BA, Chatterjee K (2006). ACC/AHA 2006 guidelines for the management of patients with valvular heart disease: a report of the american college of cardiology/ American heart association task force on practice guidelines (Writing committee to revise the 1998 guidelines for the management of patients with valvular heart disease) Developed in collaboration with the society of cardiovascular anesthesiologists endorsed by the society for cardiovascular angiography and interventions and the society of thoracic surgeons. J Am Coll Cardiol.

[9] Ling LH, Enriquez-Sarano M (2000). Long-term outcomes of patients with flail mitral valve leaflets. Coron Artery Dis.

[10] Fierro-Carrion G, Mahler DA, Ward J, Baird JC (2004). Comparison of continuous and discrete measurements of dyspnea during exercise in patients with COPD and normal subjects. Chest.

[11] Tunick PA, Freedberg RS, Gargiulo A, Kronzon I (1992). Exercise Doppler echocardiography as an aid to clinical decision making in mitral valve disease. J Am Soc Echocardiogr.

[12] Tischler MD, Battle RW, Saha M, Niggel J, LeWinter MM (1995). Observations suggesting a high incidence of exercise-induced severe mitral regurgitation in patients with mild rheumatic mitral valve disease at rest. J Am Coll Cardiol.

[13] Grigioni F, Tribouilloy C, Avierinos JF (2008). Outcomes in mitral regurgitation due to flail leaflets: a multicenter european study. J Am Coll Cardiol Img.

[14] Rosen SE, Borer JS, Hochreiter C (1994). Natural history of the asymptomatic/minimally symptomatic patient with severe mitral regurgitation secondary to mitral valve prolapse and normal right and left ventricular performance. Am J Cardiol.

[15] Enriquez-Sarano M, Avierinos J-F, Messika-Zeitoun D (2005). Quantitative determinants of the outcome of asymptomatic mitral regurgitation. N Engl J Med.

[16] Ling LH, Enriquez-Sarano M, Seward JB (1996). Clinical outcome of mitral regurgitation due to flail leaflet. N Engl J Med.

[17] Rosenhek R, Rader F, Klaar U (2006). Outcome of watchful waiting in asymptomatic severe mitral regurgitation. Circulation.

[18] Stoddard MF, Prince CR, Dillon S (1995). Exercise-induced mitral regurgitation is a predictor of morbid events in subjects with mitral valve prolapse. J Am Coll Cardiol.

[19] Starling MR, Kirsh MM, Montgomery DG, Gross MD (1993). Impaired left ventricular contractile function in patients with long-term mitral regurgitation and normal ejection fraction. J Am Coll Cardiol.

[20] Carabello BA, Nolan SP, McGuire LB (1981). Assessment of preoperative left ventricular function in patients with mitral regurgitation: value of the end-systolic wall stress-end-systolic volume ratio. Circulation.

[21] Paulsen WJ, Boughner DR, Friesen A, Persaud JA (1979). Ventricular response to isometric and isotonic exercise. Echocardiographic assessment. Br Heart J.

[22] Lee R, Haluska B, Leung DY (2005). Functional and prognostic implications of left ventricular contractile reserve in patients with asymptomatic severe mitral regurgitation. Heart (British Cardiac Society).

[23] Leung DY, Griffin BP, Stewart WJ (1996). Left ventricular function after valve repair for chronic mitral regurgitation: predictive value of preoperative assessment of contractile reserve by exercise echocardiography. J Am Coll Cardiol.

[24] Hochreiter C, Niles N, Devereux RB, Kligfield P, Borer JS (1986). Mitral regurgitation: relationship of noninvasive descriptors of right and left ventricular performance to clinical and hemodynamic findings and to prognosis in medically and surgically treated patients. Circulation.

[25] Armstrong GP, Griffin BP (2000). Exercise echocardiographic assessment in severe mitral regurgitation. Coron Artery Dis.

[26] Schiller N (1994). Exercise Doppler echocardiography in valvular and noncoronary myocardial disease. Interventional echocardiography: transesophageal, pharmacologic and intravascular. ACC Learning Center Highlights.

[27] Abramson SV, Burke JF, Kelly JJ Jr (1992). Pulmonary hypertension predicts mortality and morbidity in patients with dilated cardiomyopathy. Ann Intern Med.

[28] Enriquez-Sarano M, Rossi A, Seward JB, Bailey KR, Tajik AJ (1997). Determinants of pulmonary hypertension in left ventricular dysfunction. J Am Coll Cardiol.

[29] Cuocolo A, Sax FL, Brush JE (1990). Left ventricular hypertrophy and impaired diastolic filling in essential hypertension. Diastolic mechanisms for systolic dysfunction during exercise. Circulation.

[30] Genovesi-Ebert A, Marabotti C, Palombo C (1994). Echo Doppler diastolic function and exercise tolerance. Int J Cardiol.

[31] Kim HK, Kim YJ, Chung JW (2004). Impact of left ventricular diastolic function on exercise capacity in patients with chronic mitral regurgitation: an exercise echocardiography study. Clin Cardiol.

[32] Leier CV, Binkley PF, Starling RC, Huss-Randolph P (1989). Disparity between improvement in left ventricular function and changes in clinical status and exercise capacity during chronic enoximone therapy. Am Heart J.

[33] Okura H, Inoue H, Tomon M (2000). Impact of Doppler-derived left ventricular diastolic performance on exercise capacity in normal individuals. Am Heart J.

[34] Otsuji Y, Handschumacher MD, Schwammenthal E (1997). Insights from three-dimensional echocardiography into the mechanism of functional mitral regurgitation: direct in vivo demonstration of altered leaflet tethering geometry. Circulation.

[35] Hung J, Otsuji Y, Handschumacher MD, Schwammenthal E, Levine RA (1999). Mechanism of dynamic regurgitant orifice area variation in functional mitral regurgitation: physiologic insights from the proximal flow convergence technique. J Am Coll Cardiol.

[36] Levine RA (2004). Dynamic mitral regurgitation--more than meets the eye. N Engl J Med.

[37] Yiu SF, Enriquez-Sarano M, Tribouilloy C, Seward JB, Tajik AJ (2000). Determinants of the degree of functional mitral regurgitation in patients with systolic left ventricular dysfunction: A quantitative clinical study. Circulation.

[38] He S, Fontaine AA, Schwammenthal E, Yoganathan AP, Levine RA (1997). Integrated mechanism for functional mitral regurgitation: leaflet restriction versus coapting force: *in vitro* studies. Circulation.

[39] Levine RA, Hung J (2003). Ischemic mitral regurgitation, the dynamic lesion: clues to the cure. J Am Coll Cardiol.

[40] Lapu-Bula R, Robert A, Van Craeynest D (2002). Contribution of exercise-induced mitral regurgitation to exercise stroke volume and exercise capacity in patients with left ventricular systolic dysfunction. Circulation.

[41] Lebrun F, Lancellotti P, Pierard LA (2001). Quantitation of functional mitral regurgitation during bicycle exercise in patients with heart failure. J Am Coll Cardiol.

[42] Pierard LA, Lancellotti P (2004). The role of ischemic mitral regurgitation in the pathogenesis of acute pulmonary edema. N Engl J Med.

[43] Peteiro J, Freire E, Montserrat L, Castro-Beiras A (1998). The effect of exercise on ischemic mitral regurgitation. Chest.

[44] Izumi S, Miyatake K, Beppu S (1987). Mechanism of mitral regurgitation in patients with myocardial infarction: a study using real-time two-dimensional Doppler flow imaging and echocardiography. Circulation.

[45] Kono T, Sabbah HN, Rosman H (1992). Left ventricular shape is the primary determinant of functional mitral regurgitation in heart failure. J Am Coll Cardiol.

[46] Lancellotti P, Lebrun F, Pierard LA (2003). Determinants of exercise-induced changes in mitral regurgitation in patients with coronary artery disease and left ventricular dysfunction. J Am Coll Cardiol.

[47] Giga V, Ostojic M, Vujisic-Tesic B (2005). Exercise-induced changes in mitral regurgitation in patients with prior myocardial infarction and left ventricular dysfunction: relation to mitral deformation and left ventricular function and shape. Eur Heart J.

[48] Barzilai B, Davis VG, Stone PH, Jaffe AS (1990). Prognostic significance of mitral regurgitation in acute myocardial infarction. The MILIS Study Group. Am J Cardiol.

[49] Feinberg MS, Schwammenthal E, Shlizerman L (2000). Prognostic significance of mild mitral regurgitation by color Doppler echocardiography in acute myocardial infarction. Am J Cardiol.

[50] Lamas GA, Mitchell GF, Flaker GC (1997). Clinical significance of mitral regurgitation after acute myocardial infarction. Survival and Ventricular Enlargement Investigators. Circulation.

[51] Lancellotti P, Troisfontaines P, Toussaint AC, Pierard LA (2003). Prognostic importance of exercise-induced changes in mitral regurgitation in patients with chronic ischemic left ventricular dysfunction. Circulation.

[52] Lancellotti P, Gerard PL, Pierard LA (2005). Long-term outcome of patients with heart failure and dynamic functional mitral regurgitation. Eur Heart J.

[53] Peteiro J, Monserrrat L, Bouzas A (2006). Prognostic value of mitral regurgitation assessment during exercise echocardiography in patients with known or suspected coronary artery disease. J Am Soc Echocardiogr.

[54] Trichon BH, Felker GM, Shaw LK, Cabell CH, O'Connor CM (2003). Relation of frequency and severity of mitral regurgitation to survival among patients with left ventricular systolic dysfunction and heart failure. Am J Cardiol.

[55] Takano H, Adachi H, Ohshima S, Taniguchi K, Kurabayashi M (2006). Functional mitral regurgitation during exercise in patients with heart failure. Circ J.

[56] Abraham WT, Fisher WG, Smith AL (2002). Cardiac resynchronization in chronic heart failure. N Engl J Med.

[57] Bristow MR, Saxon LA, Boehmer J (2004). Cardiac-resynchronization therapy with or without an implantable defibrillator in advanced chronic heart failure. N Engl J Med.

[58] Cleland JG, Daubert JC, Erdmann E (2005). The effect of cardiac resynchronization on morbidity and mortality in heart failure. N Engl J Med.

[59] Nishimura RA, Hayes DL, Holmes DR, Tajik J (1995). Mechanism of hemodynamic improvement by dual-chamber pacing for severe left ventricular dysfunction: An acute Doppler and catheterization hemodynamic study. J Am Coll Cardiol.

[60] Auricchio A, Stellbrink C, Block M (1999). Effect of Pacing Chamber and atrioventricular delay on acute systolic function of paced patients with congestive heart failure. Circulation.

[61] Kass DA, Chen C-H, Curry C (1999). Improved left ventricular mechanics from acute VDD pacing in patients with dilated cardiomyopathy and ventricular conduction delay. Circulation.

[62] Soliman OII, Geleijnse ML, Theuns DAMJ (2008). Reverse of left ventricular volumetric and structural remodeling in heart failure patients treated with cardiac resynchronization therapy. Am J Cardiol.

[63] Brandt RR, Reiner C, Arnold R (2006). Contractile response and mitral regurgitation after temporary interruption of long-term cardiac resynchronization therapy. Eur Heart J.

[64] Ennezat PV, Gal B, Kouakam C (2006). Cardiac resynchronisation therapy reduces functional mitral regurgitation during dynamic exercise in patients with chronic heart failure: an acute echocardiographic study. Heart (British Cardiac Society).

[65] Lancellotti P, Melon P, Sakalihasan N (2004). Effect of cardiac resynchronization therapy on functional mitral regurgitation in heart failure. Am J Cardiol.

[66] Madaric J, Vanderheyden M, Van Laethem C (2007). Early and late effects of cardiac resynchronization therapy on exercise-induced mitral regurgitation: relationship with left ventricular dyssynchrony, remodelling and cardiopulmonary performance. Eur Heart J.

[67] Ypenburg C, Lancellotti P, Tops LF (2007). Acute effects of initiation and withdrawal of cardiac resynchronization therapy on papillary muscle dyssynchrony and mitral regurgitation. J Am Coll Cardiol.

[68] Kanzaki H, Bazaz R, Schwartzman D (2004). A mechanism for immediate reduction in mitral regurgitation after cardiac resynchronization therapy: Insights from mechanical activation strain mapping. J Am Coll Cardiol.

[69] Ennezat PV, Marechaux S, Le Tourneau T (2006). Myocardial asynchronism is a determinant of changes in functional mitral regurgitation severity during dynamic exercise in patients with chronic heart failure due to severe left ventricular systolic dysfunction. Eur Heart J.

[70] D'Andrea A, Caso P, Cuomo S (2007). Effect of dynamic myocardial dyssynchrony on mitral regurgitation during supine bicycle exercise stress echocardiography in patients with idiopathic dilated cardiomyopathy and 'narrow' QRS. Eur Heart J.

[71] Breithardt OA, Sinha AM, Schwammenthal E (2003). Acute effects of cardiac resynchronization therapy on functional mitral regurgitation in advanced systolic heart failure. J Am Coll Cardiol.

[72] Pierard LA (2007). Left ventricular dyssynchrony and functional mitral regurgitation: two dynamic conditions. Eur Heart J.

